# Overexpression of OsHox32 Results in Pleiotropic Effects on Plant Type Architecture and Leaf Development in Rice

**DOI:** 10.1186/s12284-016-0118-1

**Published:** 2016-09-13

**Authors:** Ying-ying Li, Ao Shen, Wei Xiong, Qiong-lin Sun, Qian Luo, Ting Song, Zheng-long Li, Wei-jiang Luan

**Affiliations:** College of Life Sciences, Tianjin Key Laboratory of Animal and Plant Resistance, Tianjin Normal University, Tianjin, 300387 People’s Republic of China

**Keywords:** Rice (*Oryza sativa* L.), OsHox32, Overexpression, Plant type, Rolled leaf

## Abstract

**Background:**

The Class III homeodomain Leu zipper (HD-Zip III) gene family plays important roles in plant growth and development. Here, we analyze the function of *OsHox32*, an HD-Zip III family member, and show that it exhibits pleiotropic effects on regulating plant type architecture and leaf development in rice.

**Results:**

Transgenic lines overexpressing *OsHox32* (*OsHox32-OV*) produce narrow leaves that roll towards the adaxial side. Histological analysis revealed a decreased number of bulliform cells in *OsHox32-OV* lines. In addition, the angle between the leaf and culm was reduced, resulting in an erect plant phenotype. The height of the plants was reduced, resulting in a semi-dwarf phenotype. In addition, the chlorophyll level was reduced, resulting in a decrease in the photosynthetic rate, but water use efficiency was significantly improved, presumably due to the rolled leaf phenotype. *OsHox32* exhibited constitutive expression in different organs, with higher mRNA levels in the stem, leaf sheath, shoot apical meristems and young roots, suggesting a role in plant-type and leaf development. Moreover, *OsHox32* mRNA levels were higher in light and lower in the dark under both long-day and short-day conditions, indicating that *OsHox32* may be associated with light regulation. Photosynthesis-associated and chlorophyll biosynthesis-associated genes were down-regulated to result in the reduction of photosynthetic capacity in *OsHox32-OV* lines. mRNA level of six rice *YABBY* genes is up-regulated or down-regulated by *OsHox32*, suggesting that *OsHox32* may regulate the architecture of plant type and leaf development by controlling the expression of *YABBY* genes in rice. In addition, *OsHox32* mRNA level was induced by the phytohormones, indicating that *OsHox32* may be involved in phytohormones regulatory pathways.

**Conclusions:**

*OsHox32*, an HD-Zip III family member, plays pleiotropic effects on plant type architecture and leaf development in rice.

**Electronic supplementary material:**

The online version of this article (doi:10.1186/s12284-016-0118-1) contains supplementary material, which is available to authorized users.

## Background

The functions of a leaf, including photosynthesis, respiration and transpiration, are critical for plant survival and are dependent on three-dimensional architecture specific to the plant type (Govaerts et al. 1996). Leaf shape and morphological architecture are considered the most important agronomic traits in rice. Moderate leaf rolling in rice can improve its light capture and gas exchange abilities (Eshed et al. 2001; Moon and Hake. 2011); in addition, appropriate leaf rolling is also related to improved stress responses via reduced direct solar radiation exposure and decreased leaf transpiration under drought stress (Lang et al. 2004; Zhang et al. 2009). Therefore, moderate leaf rolling is highly important for increased grain yield in rice. Recently, several genes regulating the leaf rolling phenotype have been identified and characterized in rice. For example, SHALLOT-LIKE1 (SLL1)/RL9, a transcription factor of the KANADI family, regulates leaf abaxial cell development in rice (Yan et al. 2008; Zhang et al. 2009). *sll1* mutants display extremely incurved leaves due to the defective development of sclerenchymatous cells on the abaxial side of the leaf; moreover, the overexpression of *SLL1* also resulted in leaf rolling by stimulating phloem development on the abaxial side and suppressing bulliform cell and sclerenchyma development on the adaxial side (Zhang et al. 2009). *Adaxialized Leaf 1* (*ADL1*) encodes a plant-specific calpain-like Cys protease and is required for the establishment of the adaxial-abaxial axis in the leaf primordium (Hibara et al. 2009). *adl1* mutants display abaxially rolled leaves due to the increase of bulliform cells on the adaxial side and the formation of bulliform-like cells on the abaxial side of the leaf (Hibara et al. 2009). The overexpression of *Abaxially Curled Leaf1* (*ACL1*) and its homolog *ACL2* results in abaxial leaf curling due to increased bulliform cell number and size in rice (Li et al. 2010). *Rice outermost cell-specific gene5* (*Roc5*), a member of homeodomain leucine zipper class IV, also controls leaf rolling by regulating bulliform cell fate and development (Zou et al. 2011). The ectopic expression of *Roc5* results in adaxially rolled leaves, whereas cosuppression of *Roc5* results in abaxial leaf rolling (Zou et al. 2011). Moreover, several cellulose synthase-like genes and glycosylphosphatidylinositol-anchored proteins have been found to control leaf rolling in rice. The *narrow leaf and dwarf1* (*ND1*)/the *curled and dwarf leaf 1* (*OsCD1*)/the *narrow and rolled leaf1* (*OsCslD4*) encodes a member of the cellulose synthase-like D subfamily. Defects in *ND1/OsCD1/OsCslD4* produced dwarfed plants with narrow and rolled leaves due to changes in cell wall composition (Li et al. 2009; Hu et al. 2010; Luan et al. 2011). Furthermore, SEMI-ROLLED LEAF1 (SRL1), a glycosylphosphatidylinositol-anchored protein, was found to regulate the formation of bulliform cells in the adaxial cell layers, leading to leaf rolling in rice (Xiang et al. 2012). Recently, a zinc finger homeodomain class homeobox transcription factor (*OsZHD1*) was found in rice to induce abaxially curling and drooping due to increased bulliform cell numbers (Xu et al. 2014).

Homeobox genes were originally found in *Drosophila* to control the development of body segments (Scott et al. 1989). Thus far, they have been identified in different organisms, including various animal species, yeast, fungi, and higher plants. Homeobox genes contain a conserved DNA-binding motif known as the homeodomain that consists of 60 amino acid residues. In higher plants, a homeodomain superfamily with a closely linked leucine zipper motif, named HD-Zip, was first discovered in *Arabidopsis thaliana* (Ruberti et al. 1991). At present, HD-Zips have been identified in plants such as sunflower (Chan and Gonzalez 1994), soybean (Moon et al. 1996), carrot (Kawahara et al. 1995), tomato (Meissner and Theres 1995; Tornero et al. 1996), and rice (Meijer et al. 1997; Itoh et al. 2008; Luan et al. 2013). Based on differences of gene structure, motifs, and specific DNA binding sequence (Sessa et al. 1998), HD-Zip members can be divided into four groups, HD-Zip I through HD-Zip IV. All HD-Zip proteins function as mediators of plant development. Five members of the HD-Zip III, PHABULOSA (PHB), PHAVOLUTA (PHV), REVOLUTA (REV), CORONA (CNA), and AtHB8 in *Arabidopsis*, serve partially redundant and antagonistic roles in the development of Arabidopsis (Emery et al. 2003; Prigge et al. 2005). PHB, PHV, REV and CNA are related to the initiation of the shoot apical meristem (SAM) as well as the formation of axillary SAMs (Talbert et al. 1995; Otsuga et al. 2001; Emery et al. 2003; Green et al. 2005; Prigge et al. 2005), whereas AtHB8 is implicated in vascularization (Baima et al. 2001). In addition, *REV*, *PHB* and *PHV* can interact with *KANADI* to regulate the abaxial-adaxial patterning of lateral organs via feedback mechanisms. *KANADI* is required for the formation of abaxial tissues, but its expression represses that of *REV*, *PHB* and *PHV* (Emery et al. 2003). Recently, studies have shown that HD-Zip III members are regulated by microRNAs such as miRNA165 and miRNA166 (Kim et al. 2005; Williams et al. 2005; Mallory and Vaucheret 2006). Mutation of these two microRNAs gives rise to HD-Zip III gain-of-function phenotypes in *Arabidopsis*. In rice, the expression patterns and ectopic expression phenotypes of five HD-Zip III members, *OsHB1*-*OsHB5*, were analyzed, suggesting that they were essential for radial pattern formation during embryogenesis and the leaf initiation process in the shoot apical meristem (Itoh et al. 2008). However, they only analyzed the effect of ectopic expression of *OsHB1*, *OsHB3*, and *OsHB5* in Itoh et al.’ study, but not *OsHB4* (*OsHox32*). In this study, we analyzed the pleiotropic functions of *OsHox32* in the development and architecture of rice plant type and leaf. Using reverse genetics, we demonstrate that overexpression of *OsHox32* leads to leaf rolling and semi-dwarf phenotype in rice.

## Results

### *OsHox32* is a Member of the HD-Zip III Family

**Fig. 1:**
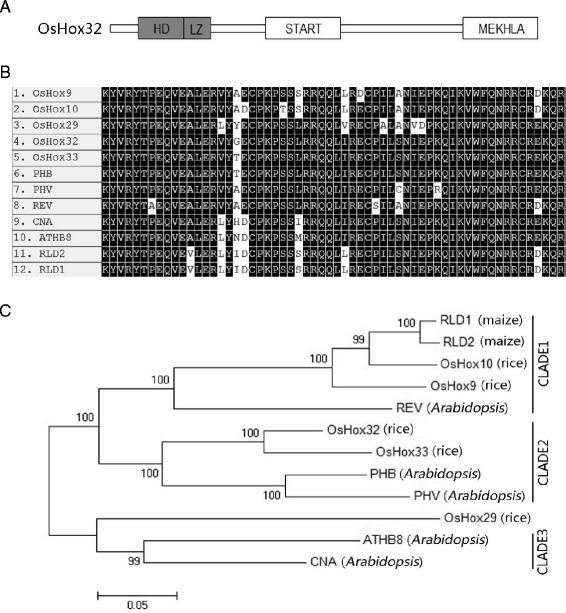
HD-Zip III family members in rice and cross-species sequence analysis. **a** Domain composition of the OsHox32 protein; HD: Homeodomain; LZ: leucine-zipper domain; START domain and MEKHLA domain. **b** Conserved homeodomain amino acid sequence for OsHox32 and other HD-Zip members in different species identified by BLAST homology. Sequences were aligned using MEGA software. **c** Phylogenetic tree of 12 HD-Zip III proteins from three plant species: *Oryza sativa* (Os), *Arabidopsis thaliana* (AT), and *Zea mays* (Zm); the protein sequence accession numbers are OsHox9: Q9AV49; OsHox10: Q6TAQ6; OsHox29: Q5QMZ9; OsHox32: NP_001050745; OsHox33: NP_001067260; PHB: BAJ14107.1; PHV: NP_174337.1; REV: AAF42938.1; CNA: NP_175627.1; ATHB8: NP_195014.1; RLD1: NP_001105533.1; and RLD2: NP_001105994.1

*OsHox32* encodes a protein of 859 amino acid residues. Analysis of protein sequence revealed that it belongs to the HD-Zip III family member (Agalou et al. 2008) and contains four conserved HD-Zip III family domains: the homeodomain (HD), the basic leucine-zipper domain (LZ), the START domain, and the MEKHLA domain (Fig. 1a). The homeodomain is highly conserved in plants and usually consists of 60 amino acid residues, 44 of which are highly conserved in rice, Arabidopsis and maize (Fig. 1b). In rice, there are five HD-Zip III members: *OsHox9*, *OsHox10*, *OsHox29*, *OsHox32*, and *OsHox33*. Phylogenetic analysis in several species shows that they are divided into three clades (Fig. 1c). *OsHox9* and *OsHox10* are more similar to the two maize (*Zea mays*) members *RLD1* and *RLD2*, which play an important role in establishing the adaxial/abaxial polarity of the maize leaf. Semi-dominant mutation of *RLD1* causes the adaxialization or partial reversal of leaf polarity and leads to the rolled leaf phenotype in maize (Juarez et al. 2004). *OsHox32* and *OsHox33* are orthologous to both Arabidopsis *PHB* and *PHV* and are grouped into clade 2. *OsHox29* and two Arabidopsis members *ATHB8* and *CNA* form clade 3.

### Overexpression of *OsHox32* Affects the Architecture of the Rice Leaf and Plant Type

**Fig. 2:**
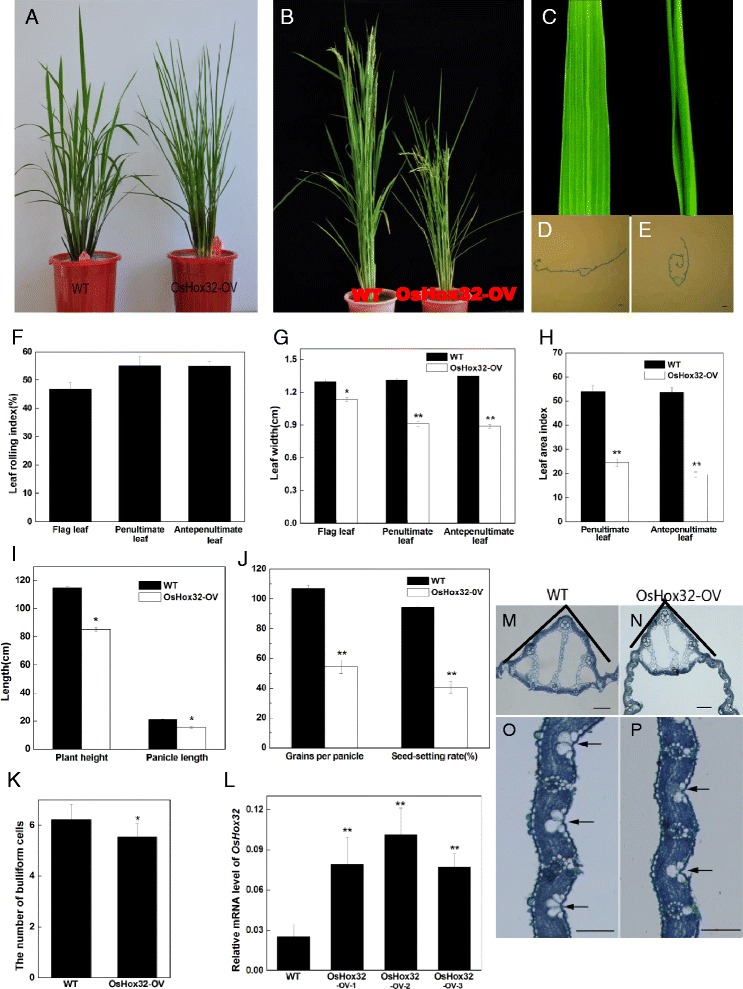
Phenotypes and histological analysis of transgenic plants overexpressing *OsHox32*. **a**–**c** Phenotypes of *OsHox32-OV* and wild type plants. **a** and **c** the tillering stage; **b** the heading stage; Wild type is at left and *OsHox32-OV* at right. **d** and **e** Cross sections of the leaves of wild type (**d**) and *OsHox32-OV* plants (**e**). **f** Index of leaf rolling (LRI) in *OsHox32-OV* plants. LRI(%) = (Lw-Ln)/Lw × 100 %. Ln, leaf width under natural rolling conditions; Lw, unfolding from rolling. **g** Width of leaves in *OsHox32-OV* and wild type plants. **h** Leaf area index in *OsHox32-OV* and wild type plants. **i** Plant height and panicle length in *OsHox32-OV* and wild type plants. **j** Grains per panicle and Seed-setting rate in *OsHox32-OV* and wild type plants. **k** Number of bulliform cells in *OsHox32-OV* and wild type plants (*n* = 30 groups). **l** qRT-PCR analysis of the *OsHox32-OV* lines. *OsHox32-OV 1*, *2* and *3* are three independent lines. **m** and **n** Cross sections of the midribs of wild type and *OsHox32-OV* plants. The black lines demarcate the angle of the midrib. **o** and **p** Bulliform cells in the leaves of wild type and *OsHox32-OV* plants. Arrows denote bulliform cells. Scale bars: 400 μm; * denotes *t* test at 0.05 significance probability level; ** denotes *t* test at 0.01 significance probability level. The *OsHox32-OV* line presented in all figures is *OsHox32-OV2*. All data are given as the mean ± SD. (*n* =10 individuals)

To reveal the function of *OsHox32* in rice, we constructed an overexpression vector for *OsHox32* and introduced it into wild type plants. We obtained 36 T_0_ transgenic plants of six independent lines. Four of the six transgenic lines displayed narrow rolled leaves that roll toward the adaxial side and assume a columnar shape (Fig. 2a–c). We selected the four lines displaying this phenotype to obtain T_2_ generation plants. All T_2_ plants produced the narrow and rolled leaf phenotype, suggesting that the phenotype is stably transmitted to offspring. The index of leaf rolling was 47, 55 and 55 % in the flag leaf, the penultimate leaf and the antepenultimate leaf, respectively, in the transgenic lines (Fig. 2f). We also measured the width of the leaf and the leaf area index in wild type and transgenic plants overexpressing *OsHox32* (*OsHox32-OV*), and found that the width in *OsHox32-OV* lines is significantly narrower for all leaf types (Fig. 2g). The leaf area index is also significantly reduced in *OsHox32-OV* lines (Fig. 2h). Additionally, the leaves were erect and the angles between leaf and culm were reduced (Fig. 2a, b). In addition, the height of the plants and the length of the panicles were reduced in *OsHox32-OV* lines compared with the wild type, producing a semi-dwarf phenotype (Fig. 2b, i). Grains per panicle and seeds setting were also reduced in *OsHox32-OV* lines compared with the wild type (Fig. 2j). We also analyzed the phenotype of *OsHox32-OV* lines using histology. In cross sections, we can see that two sides of leaf roll to the adaxial side along the midrib (Fig. 2c, d, e), and the angles of midrib and the two lateral blades were reduced in *OsHox32-OV* lines (Fig. 2m, n). Bulliform cells were thought to associate with leaf rolling (Hibara et al. 2009; Li et al. 2010; Zou et al. 2011). They are large, bubble-shaped and specialized epidermal cells on the adaxial leaf blade surface in monocotyledonous (Jane and Chiang 1991). Loss of turgor pressure or shrinkage in bulliform cells causes leaf blade to curl or roll up in grasses (Moore and Clark 1998; Kadioglu and Terzi 2007). Therefore, we accounted the number of bulliform cells in the *OsHox32-OV* lines and wild type plants, and found that the number of bulliform cells was significantly reduced in the *OsHox32-OV* lines compared with wild type plants (Fig. 2k, o, p). To confirm *OsHox32* overexpression, we randomly selected three lines displaying rolled and narrow leaves for the detection of *OsHox32* mRNA level by real-time PCR. The result of this analysis showed that the expression of *OsHox32* in transgenic lines was significantly elevated compared with wild type plants (Fig. 2l), suggesting that the overexpression constructs were efficient. Among three detected transgenic lines, the expression of *OsHox32* in *OsHox32-OV2* lines was the highest, therefore, we used *OsHox32-OV2* lines to further investigate the phenotypic and related data. Moreover, we detected *OsHox32* expression in transgenic lines without phenotypes, but the result showed that the *OsHox32* mRNA level was similar to that observed in wild type plants (data not shown). These results suggest that the phenotype of the *OsHox32-OV* lines is caused by the overexpression of *OsHox32*.

### Overexpression of *OsHox32* Affects Photosynthesis Indexes and Water use Efficiency

**Fig. 3:**
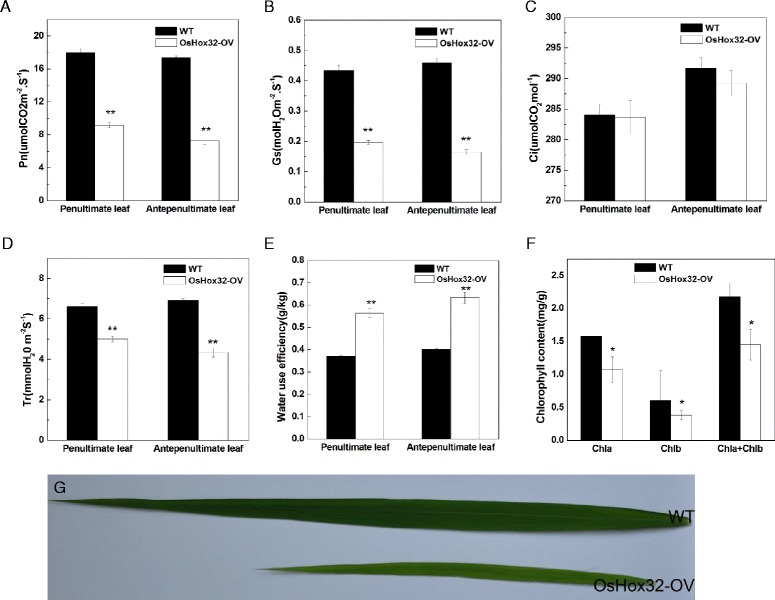
Photosynthesis indexes and chlorophyll content in *OsHox32-OV* transgenic plants. Net photosynthetic rate (Pn) (**a**), stomatal conductance (Gs) (**b**), intercellular CO_2_ concentration (Ci) (**c**), transpiration rate (Tr) (**d**), water use efficiency (**e**) and chlorophyll content (**f**) were measured in the penultimate and antepenultimate leaves of booting stage rice. All data are given as the mean ± SD (*n* =10 individuals). g Leaf color of wild type and *OsHox32-OV* plants (the flag leaves were exhibited in this figure). WT is wild type Nipponbare; OsHox32-OV is *OsHox32-OV2*. Chla, Chlb and Chla + Chlb are chlorophyll A, chlorophyll B and total chlorophyll, respectively

We measured photosynthesis indexes and water use efficiency to further characterize the effect of the rolled leaf phenotype in *OsHox32-OV* transgenic plants. In the booting stage, net photosynthetic rate (Pn), stomatal conductance (Gs), intercellular CO_2_ concentration (Ci), transpiration rate (Tr) and water use efficiency (WUE) were measured in *OsHox32-OV* and wild type plants. Our results showed that Pn, Gs and Tr were significantly reduced, whereas WUE was significantly improved in the penultimate and antepenultimate leaves of *OsHox32-OV* plants compared with wild type plants (Fig. 3a, b, d, and e), suggesting that the rolled leaf phenotype in *OsHox32-OV* lines affects stomatal conductance and reduces transpiration to improve WUE. However, Ci was not significantly altered between *OsHox32-OV* and wild type plants (Fig. 3c), suggesting that the reduction of Pn in *OsHox32-OV* plants is not the result of the change of stomatal function. Because *OsHox32-OV* plants exhibit laurel-green leaves (Fig. 3g), we assumed that their chlorophyll levels may be reduced. We therefore measured the chlorophyll content in the leaves of *OsHox32-OV* and wild type plants. This analysis showed that the chlorophyll A and chlorophyll B contents were significantly reduced in *OsHox32-OV* plants (Fig. 3f), indicating that the decrease in chlorophyll level led to the reduction of the photosynthetic capacity of *OsHox32-OV* lines.

### The Expression Pattern of *OsHox32*

**Fig. 4:**
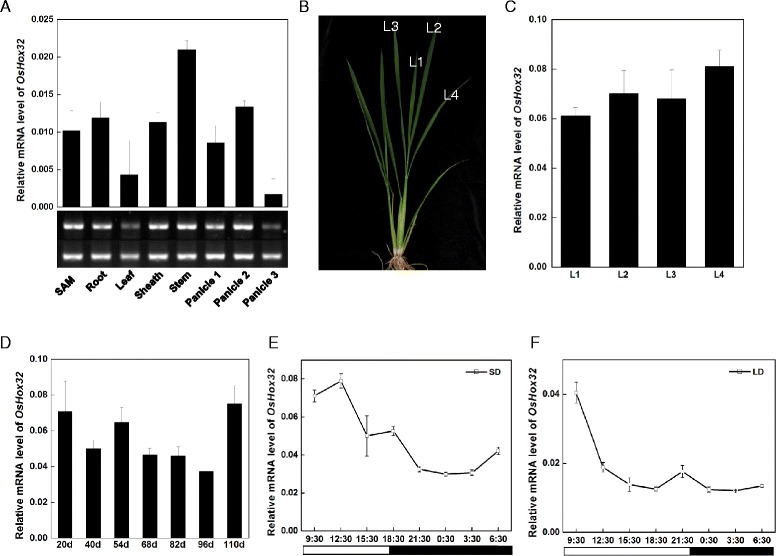
Expression analysis of *OsHox32*. **a** *OsHox32* expression in different organs. SAM: shoot apical meristem. Root indicates the young roots of a 30-day-old seedling. Leaf indicates a mature leaf blade at the late tillering stage (about 12:00 at noon). Sheath indicates a leaf sheath at the late tillering stage. Stem indicates a stem at the first internode. Panicles 1–3 represent the different stages of young panicles; panicle 1, 13 cm long young panicle; panicle 2, 4 cm long young panicle; panicle 3, the panicle before flowering (heading stage). **b** and **c** *OsHox32* expression in leaves of different ages. **b** a wild type plant at 60 days old in the field. L1-L4 are leaves of different ages. **d** *OsHox32* expression at different developmental stages. The penultimate leaves were collected at different development stages to analyze the expression of *OsHox32.* **e** and **f** *OsHox32* expression during different photoperiods. After treatment with different photoperiods, RNA was isolated from leaves once every 3 h for 24 h to analyze the expression of *OsHox32* by qRT-PCR. **e**, Expression of *OsHox32* under SD conditions. **f** Expression of *OsHox32* under LD conditions. Black filled bars correspond to dark stage and open bars correspond to light stage

To analyze *OsHox32* expression in different organs, we isolated RNA from shoot apical meristems (SAM), young roots, mature leaves, leaf sheathes, stem of the first internode, and different stages of panicles (panicle 1–3). qRT-PCR showed that *OsHox32* presented different expression levels in these organs. Higher *OsHox32* expression levels were detected in the stem, leaf sheathes, SAM, young roots and young panicles, whereas *OsHox32* expression was low in mature leaves and in the panicle before flowering (Fig. 4a), similar to online microarray data (RiceXPro, ricexpro.dna.affrc.go.jp/). We also analyzed *OsHox32* expression in different leaf-aged leaves, and the result showed that *OsHox32* expression is not significant difference among different ages of leaves (Fig. 4b, c). In addition, we analyzed the expression of *OsHox32* during different growth stages in the field and found that the *OsHox32* transcript is not differentially expressed at different development stages, but was slightly increased in the seedling stage (20d) and the flowering stage (110d) (Fig. 4d). To investigate whether *OsHox32* transcript level is affected by the circadian clock, we examined the *OsHox32* mRNA level at different photoperiods over the course of 24 h. The result showed that *OsHox32* exhibited higher expression during light stages and lower expression during dark stages under both long-day and short-day conditions, similar to the microarray data (RiceXPro, ricexpro.dna.affrc.go.jp/) (Fig. 4e, f). The peak of *OsHox32* transcript expression occurred 1.5 h after dawn under long-day conditions, but the peak occurred at 4.5 h after dawn under short-day conditions.

### Subcellular Localization of *OsHox32*

**Fig. 5:**
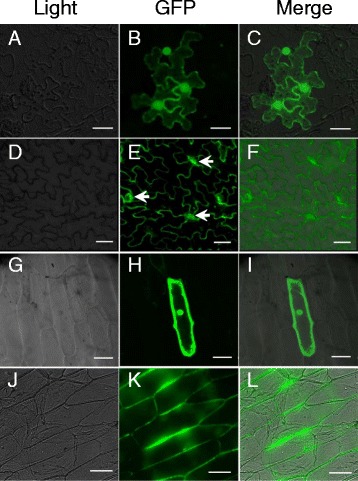
Transient expression of OsHox32 in tobacco and onion epidermal cells. **a**–**f** Subcellular localization of *OsHox32* in tobacco epidermal cells. **g**–**l** Subcellular localization of *OsHox32* in onion epidermal cells. **a**–**c** and **g**–**i**: GFP signal from the pCAMBIA35S::GFP empty vector. **d**–**f** and **j**–**l** GFP signal from the pCAMBIA35S::OsHox32-GFP fusion vector. Scale bars: 50 μm. arrows indicate stoma

To further analyze the function of *OsHox32*, we constructed a vector encoding a fusion of the *OsHox32* gene and green fluorescent protein (GFP) to investigate the subcellular localization of *OsHox32*. The correctly fused pCAMBIA35S::OsHox32-GFP vector and empty vector were introduced into tobacco leaves (*Nicotiana tabacum*) to produce transient expression via infiltration of an *Agrobacterium tumefaciens* strain. We found that the OsHox32-GFP signal was observed in the periphery of tobacco epidermal cells and stomata (Fig. 5d–f). We suspected that *OsHox32* would be localized in the cytoplasm, which in mature plant epidermal cells is restricted to the cell periphery due to a large central vacuole. The GFP signal generated by the empty vector was detected nonspecifically throughout the cell, including in the cytoplasm and nucleus (Fig. 5a–c). Meanwhile, transient expression of pCAMBIA35S::OsHox32-GFP vector and empty vector in onion (*Allium cepa*) epidermal cells via particle bombardment showed that OsHox32-GFP signal was located in the periphery (Fig. 5j–l). In contrast, the GFP signal from the empty vector was detected nonspecifically throughout the entire cell (Fig. 5g–i). The results of these transient expression methods were consistent, suggesting that *OsHox32* may function in the cytoplasm and stomata.

### Effect of Phytohormone Signal on *OsHox32* Expression

**Fig. 6:**
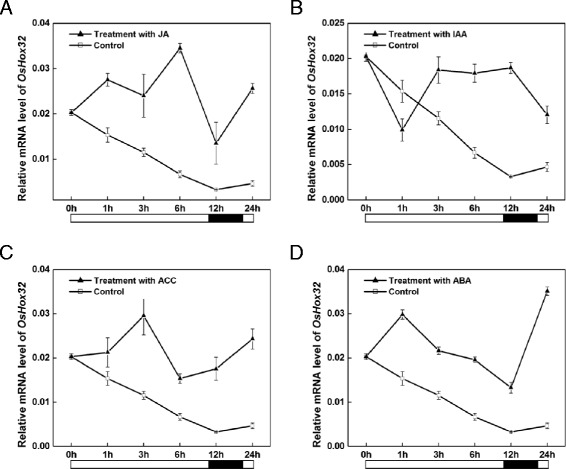
Effect of phytohormone signal on *OsHox32* expression. **a**–**d** qRT- PCR analysis of *OsHox32* expression after treatment with JA, IAA, ACC and ABA, respectively. 4-week-old wild type seedlings were sprayed with JA, IAA, ACC and ABA, respectively. Water treatment was used as a control. RNA was isolated from leaves to analyze the expression of *OsHox32* by qRT- PCR. Black filled bars indicate the dark stage, and open bars indicate the light stage

Previous studies have shown that HD-ZipIII members are associated with auxin response (Baima et al. 1995; Itoh et al. 2008). To investigate whether *OsHox32* responds to phytohormone signals, we treated wild-type plants with indole-3-acetic acid (IAA), jasmonic acid (JA), 1-Aminocyclopropanecarboxylic acid (ACC) and abscisic acid (ABA) and subsequently detected the expression of *OsHox32* by real-time PCR. The results showed that the expression of *OsHox32* was up-regulated following JA, ACC, ABA and IAA treatment, suggesting that the expression of *OsHox32* can be induced by JA, ACC, ABA and IAA (Fig. 6a–d). The transcription of *OsHox32* displays similar patterns after treatment with JA, ACC and ABA, with peaks during light and the lows during the dark (Fig. 6a, c, and d). Moreover, two peaks were observed. The first peak occurred at 6 h, 3 h or 1 h after treatment with JA, ACC or ABA, respectively (Fig. 6a, c, and d). The second peak occurred 24 h after treatment with JA, ACC or ABA (Fig. 6a, c, and d), possibly due to the change from the light stage to dark stage. The expression pattern following IAA treatment was different from that of JA, ACC and ABA treatments. The expression of *OsHox32* was reduced at 1 h, and then was induced to a broad peak between 3 and 12 h (Fig. 6b). Interestingly, the transcription of *OsHox32* was still in much higher during the dark stage (12 h) after treatment with IAA (Fig. 6b). This suggests that IAA treatment may alter the pattern of *OsHox32* expression (ie, higher expression in light than in dark).

### Photosynthesis-Associated and Chlorophyll Biosynthesis-Associated Genes Were Down-Regulated in *OsHox32-OV* Plants

**Fig. 7:**
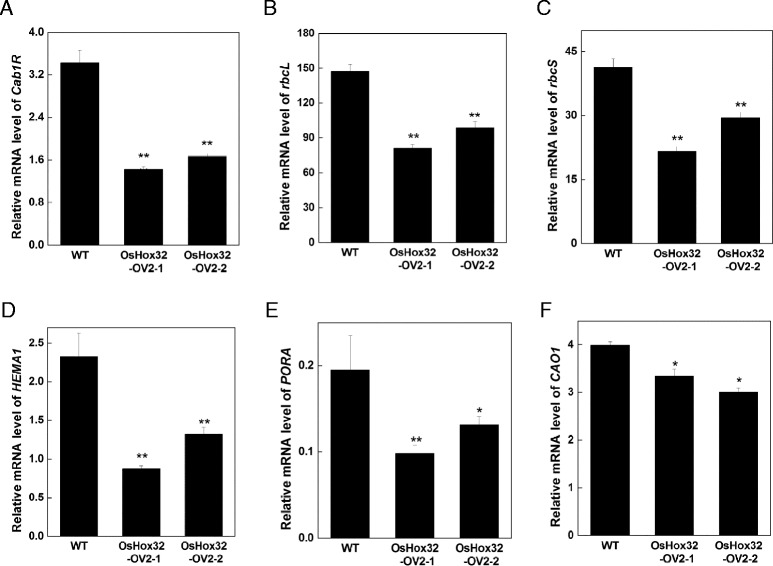
Expression of photosynthesis- and chlorophyll biosynthesis-associated genes in *OsHox32-OV* plants. RNA was isolated from the leaves of wild type and T_2_ generation *OsHox32-OV2* plants at the heading stage. qRT-PCR was used to analyze the expression of different genes in wild type and *OsHox32-OV* plants. **a**–**c** Expression of photosynthesis-associated genes; **d**–**f** Expression of chlorophyll biosynthesis-associated genes

Given the reduction in photosynthetic capacity of *OsHox32-OV* plants and higher mRNA level of *OsHox32* in the light stage, we next analyzed the expression of photosynthesis-associated and chlorophyll biosynthesis-associated genes in *OsHox32-OV* plants. Three photosynthesis-associated genes - *Cab1R* (encoding light-harvesting Chl a/b binding proteins of PSII), *rbcL* and *rbcS* (encoding the large and the small subunits of Rubisco, respectively) - were down-regulated in *OsHox32-OV* plants (Fig. 7a–c), suggesting that the reduction of photosynthetic capacity may resulted from the down-regulation of photosynthesis-associated genes in *OsHox32-OV* plants. Similarly, three chlorophyll biosynthesis-associated genes - *HEMA1* (encoding a glutamyl tRNA reductase), *PORA* (encoding a NADPH-dependent proto-chlorophyllide oxidoreductase) and *CAO1* (encoding chlorophyllide A oxygenase) - were also down-regulated in *OsHox32-OV* plants (Fig. 7d–f), indicating that chlorophyll biosynthesis may be inhibited in *OsHox32-OV* plants. These results are consistent with the phenotype of *OsHox32-OV* plants, suggesting that *OsHox32* may affect the change of photosynthetic capacity by the regulation of the expression of photosynthesis-associated and chlorophyll biosynthesis-associated genes.

### *OsHox32* Regulates the Expression of *YABBY* Genes to Control the Architecture of Plant Type and Leaf in Rice

**Fig. 8:**
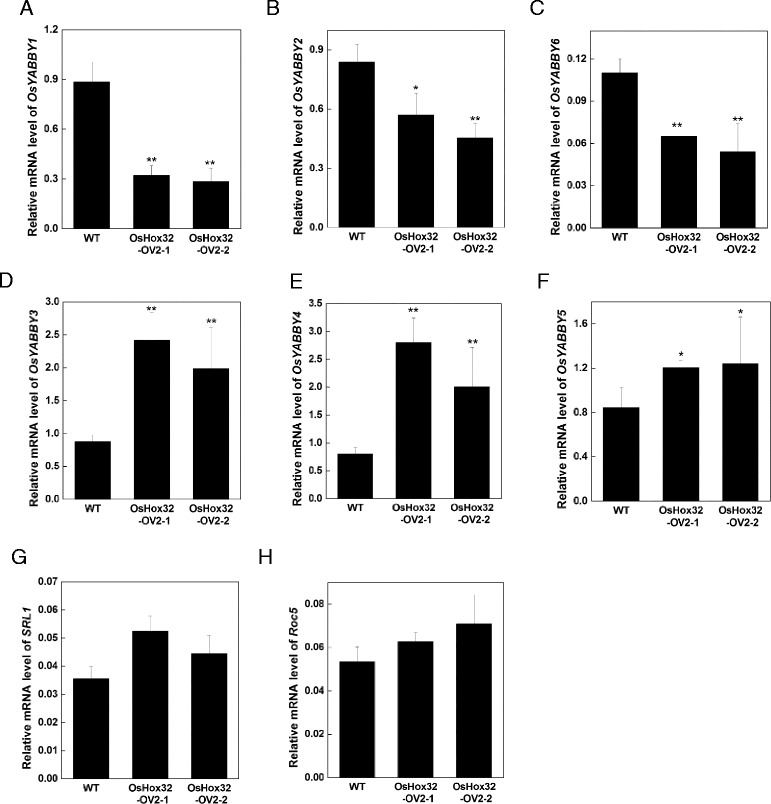
Expression of rice *YABBY* genes and rolling-associated genes in *OsHox32-OV* plants. RNA was isolated from the leaves of wild type and T_2_ generation *OsHox32-OV2* plants at the heading stage. qRT-PCR was used to analyze the expression of different genes in wild type and *OsHox32-OV* plants. **a**–**c** Expression of *OsYABBY1*, *OsYABBY2* and *OsYABBY6* respectively; **d**–**f** Expression of *OsYABBY3*, *OsYABBY4* and *OsYABBY5* respectively; **g** and **h** Expression of rolled leaf related genes

*YABBY* genes have an important role in the promotion of leaf expansion, the establishment of leaf polarity, and the general genetic program of lamina formation in *Arabidopsis thaliana* (Sawa et al. 1999; Siegfried et al. 1999; Stahle et al. 2009; Sarojam et al. 2010). In addition, Overexpression and cosuppression of *OsYABBY1* resulted in semi-dwarf and rolled leaf phenotype in rice respectively (Dai et al. 2007b). To investigate whether *OsHox32* regulates the expression of *YABBY* genes in rice, we analyzed the expression of rice *YABBY* genes in *OsHox32-OV* plants. Total 8 *YABBY* members (including *OsYABBY1* ~ *OsYABBY7* and *DL*) were found and divided into four groups (group A ~ D) in rice. *OsYABBY1*, *OsYABBY2* and *OsYABBY6* were more similar to constitute group A. *OsYABBY3*, *OsYABBY4* and *OsYABBY5* were more similar to constitute group B. *OsYABBY7* and *DL* were distinct from the other *YABBY* members to constitute group C and D (Toriba et al. 2007). In addition, from tissue-specific expression of 8 *YABBY* members, *OsYABBY1* ~ *OsYABBY6* displays high expression in leaf, however, *OsYABBY7* and *DL* were predominantly expressed in the young inflorescences and reproductive organs (Toriba et al. 2007). Therefore, we analyzed *OsYABBY1* ~ *OsYABBY6* mRNA level in *OsHox32-OV* plants by qRT-PCR. The result showed that the expression of *OsYABBY1*, *OsYABBY2* and *OsYABBY6* were significantly up-regulated, whereas *OsYABBY3*, *OsYABBY4* and *OsYABBY5* were significantly down-regulated in *OsHox32-OV* plants (Fig. 8a–f), suggesting that *OsHox32* may regulate the architecture of plant type and leaf by controlling the expression of *YABBY* genes in rice.

In addition, we analyzed the expression of two rolled leaf-related genes in *OsHox32-OV* plants and found that the expression of *Roc5* (encoding a member of homeodomain leucine zipper Class IV) and *SRL1* (encoding a glycosylphosphatidylinositol-anchored protein) did not significantly differ between *OsHox32-OV* and wild type plants (Fig. 8g, h), indicating that *OsHox32* does not regulate the expression of *Roc5* and *SRL1*.

## Discussion

### Pleiotropic Effects of *OsHox32* on the Regulation of Plant Growth and Development

Members of the Class III homeodomain-Leu zipper family play an important role in determining plant morphological architecture. In *Arabidopsis,* there are five Class III HD-Zip genes: *REV*, *PHB*, *PHV*, *CNA*, and *ATHB8*. Phenotypic analysis of loss-of-function mutants of these genes showed that they play distinct overlapping and antagonistic roles in development. *PHB*, *PHV*, and *REV* play overlapping and crucial roles during the establishment of apical bilateral symmetry and leaf abaxial/adaxial polarity (Prigge et al. 2005); *rev phv* double mutants displayed a trumpet-shaped leaf. *REV* plays an important role in the formation of the floral meristem. *rev* mutants display defective floral organs and produce sterile flowers. *CNA* and *ATHB8* exhibit antagonistic functions toward *REV* during the formation of the lateral shoot meristem and the floral meristem, which can restore the partial phenotype of *rev* mutants (Prigge et al. 2005). In maize, *RLD1* encodes an HD-ZipIII protein whose adaxial expression is defined by miRNA166-directed transcript cleavage on the abaxial side, which is an important determinant of adaxial cell fate during maize leaf development (Juarez et al. 2004). In *Populus trichocarpa*, eight HD-Zip III genes have been identified (Ko et al. 2006; Côté et al. 2010). *PRE*, a homolog of the *Arabidopsis REV* gene, is related to the initiation of the cambium and the pattern of secondary vascular tissues (Robischon et al. 2011). Another member, *PCN*, may negatively regulate secondary vascular cell differentiation (Kim et al. 2005; Du et al. 2011). In addition, *PtrHB7*, an ortholog of *AtHB8* in Arabidopsis, plays an essential role in the regulation of balanced differentiation between secondary xylem and phloem tissues during the process of secondary growth in *Populus*. These previous studies provide evidence that HD-Zip III members have evolved distinct functions and exert different pleiotropic effects on growth and development in different plants.

In rice, there are five HD-Zip class III members: *OsHox9*, *OsHox10*, *OsHox29*, *OsHox32*, and *OsHox33*, corresponding to *OsHB1-OsHB5* as described by Itoh et al. (2008). In a study by Itoh et al., the effect of ectopic expression of *OsHB1*, *OsHB3*, and *OsHB5*, but not *OsHB4* (*OsHox32*) was analyzed; overexpression of these genes resulted in abnormalities such as rolled and filamentous leaves. In this study, we investigated the role of *OsHox32*, which corresponds to *OsHB4*. Our results showed that *OsHox32* regulates the morphological architecture of the rice leaf. Overexpression of *OsHox32* resulted in narrow and upward rolling leaves. Further histological observation showed that the number of bulliform cells decreased in *OsHox32*-OV lines. This evidence suggests that *OsHox32* has a conserved function in the regulation of the morphological architecture and development of the rice leaf. However, *OsHox32* also regulates the architecture of plant type and the photosynthetic capacity in rice except the regulation of the morphological architecture of the rice leaf. Our results demonstrated that overexpression of *OsHox32* resulted in semi-dwarf phenotype. Expressed analysis found that mRNA level of six rice *YABBY* genes is up-regulated or down-regulated by *OsHox32*. This result is in agreement with exhibiting semi-dwarf phenotype of *OsYABBY1* overexpressing. In addition, our previous study revealed that *OsHox33* plays an important role in leaf senescence (Luan et al. 2013). Therefore, HD-Zip III members have evolved distinct functions except a conserved function in the regulation of the leaf architecture in rice.

### *OsHox32* may Regulate the Leaf and Plant Type Architecture by Controlling *YABBY* Genes in Rice

In Arabidopsis, the *YABBY* genes play important roles in establishment of adaxial-baxial polarity, floral organ development and lamina formation. Loss of function of *YABBY* genes results in loss of polar differentiation of abaxial cells in the lateral organs and the defect of floral organ (Sawa et al. 1999; Sarojam et al. 2010). In rice, identified *YABBY* members act as crucial function in leaf, the floral organ and vascular development and plant type architecture. Overexpression of *OsYABB1* caused semi-dwarf phenotype, whereas cosuppression of *OsYABB1* induced rolled leaf phenotype (Dai et al. 2007b). *OsYABBY3* was expressed in the leaf and floral organ primordial, playing important roles in leaf cell growth and differentiation (Dai et al. 2007a). Knockdown of *OsYABBY3* resulted in twisted and knotted leaves. *OsYABBY4* was expressed in the meristems and developing vascular tissue of rice, without an adaxial/abaxial polar expression pattern in lateral organs, suggesting that *OsYABBY4* might play an important role in vascular development (Liu et al. 2007). *OsYABBY5/TOB1* was expressed in the lateral organs primordia, but not in the meristem, playing important roles for the initiation and growth of the lemma and palea (Tanaka et al. 2012). Knockdown of *OsYABBY5* resulted in the defect of spikelet, overexpression of *OsYABBY5* induced an excess number of floral organs such as pistils, stamens, and lodicules (Tanaka et al. 2012). These previous reports showed that the up- or down-regulation of *OsYABBY* genes seriously affected leaf development and plant type architecture. Our result indicated that *OsYABBY* genes were up- or down-regulated in *OsHox32* overexpressing plants. Moreover, the overexpression of *OsHox32* resulted in rolled leaf and semi-dwarf phenotypes. Taken together the previous studies and our results, we proposed that *OsHox32* might regulate the architecture of plant type and leaf development by controlling the expression of *YABBY* genes in rice.

### The Subcellular Localization of OsHox32

Members of HD-Zip III family contain a DNA-binding motif and exhibit transcription factor activity (Ariel et al. 2007). Many transcription factors, such as *GmERF5* in soybean, *TaWRKY10* in wheat, and *OsMYB103L* in rice, were localized in the nucleus (Hernandez-Garcia et al. 2010; Wang et al. 2013; Yang et al. 2014). In addition, the transcription factor *Roc5*, which plays a role in regulating the formation of rice leaves, was also localized in the nucleus (Zou et al. 2011). However, not all transcription factors are localized in nucleus. The heat-shock transcription factor *AtHsfA6a*, which abnormally acts as a transcriptional activator of stress-responsive genes via the ABA-dependent signaling pathway, was found in the cytoplasm and nucleus simultaneously (Hwang et al. 2014). Moreover, the transcription factor *LeERF1* in tomato was also localized both in the cytoplasm and nucleus (Zhang et al. 2012). In our study, the results of transient expression showed that *OsHox32* was localized in the cell periphery, but no significant signal was detected in the nucleus. We speculate that *OsHox32* may be a cytoplasmic protein because the cytoplasm in mature plant epidermal cells is restricted to the cell periphery due to the presence of a large central vacuole. This result is supported by the prediction by PSOT software and the Rice Genome Annotation Database (http://ricegaas.dna.affrc.go.jp/).

### Effect of Phytohormone Treatments on HD-Zip III Family Gene Expression

In *Arabidopsis*, HD-Zip III gene expression patterns are correlated with auxin distribution patterns in shoot meristem and in meristematic tissues of the vasculature (McConnell et al. 2001; Otsuga et al. 2001; Emery et al. 2003; Prigge et al. 2005). Moreover, HD-Zip III proteins can promote axial cell elongation and xylem differentiation via auxin pathways (Ilegems et al. 2010). The recently reported DRN-like (DRNL) protein interacts with HD-Zip III members to control the balance of cell proliferation and differentiation in meristematic tissues via interactions with auxin signaling (Chandler et al. 2007). In rice, members of HD-Zip III family are highly expressed in the SAM flank at an early stage of leaf initiation, then disappear from the flank in the middle stage of leaf primordium development (Itoh et al. 2008). This change is similar to that of the leaf determination process, which is regulated by local auxin accumulation in the SAM. To reveal whether *OsHox32* responds to phytohormone pathways, we treated wild-type plants with IAA, JA, ACC or ABA and monitored *OsHox32* expression by real-time PCR. We found that the expression of *OsHox32* was induced by phytohormone treatment, indicating that *OsHox32* may be involved in phytohormones regulatory pathways. However, the pattern of induction varied between IAA and JA, ACC, ABA. Under normal conditions (ie, no treatments were performed), *OsHox32* exhibits higher expression during the light stage and lower expression during the dark stage due to the influence of the circadian clock. However, the pattern of *OsHox32* expression is changed after IAA treatment; the expression of *OsHox32* remained higher during the dark stage (12 h after treatment). In contrast, the pattern of *OsHox32* expression after treatment with JA, ACC, or ABA is consistent with the pattern of expression under normal conditions. These results indicate that JA, ACC and ABA have different effects on the expression of *OsHox32* compared with auxin.

### The Function of *OsHox32* in Photosynthesis

It has been reported that leaf photosynthesis is affected in several mutants with a rolled leaf phenotype. *SLL1* encodes a SHAQKYF class MYB family transcription factor (Zhang et al. 2009). *sll1* mutants display extremely rolled leaves of a deeper shade of green. The chlorophyll content and Fv/Fm responding to the photosynthetic capacity were elevated in *sll1-1* and *sll1-2* plants due to increased numbers of mesophyll cells, suggesting increased photosynthesis due to *SLL1* deficiency (Zhang et al. 2009). The photosynthetic efficiency was also improved in the *roc5*(*out1*) mutant due to an increase in the stomatal conductance and photosynthetic rate (Zou et al. 2011). Our result showed that the chlorophyll content was significantly reduced, resulting in the reduction of photosynthetic rate in *OsHox32-OV* plants. qRT-PCR experiments suggested that the expression of photosynthesis-associated and chlorophyll biosynthesis-associated genes was down-regulated in the *OsHox32-OV* line, indicating that the overexpression of *OsHox32* affects photosynthetic capacity. In addition, *OsHox32* is more highly expressed during the light stage, suggesting that *OsHox32* may be associated with the regulation of light. Taken together, we speculate that *OsHox32* may play an important role in photosynthesis.

## Conclusions

In this study, we analyze the function of *OsHox32.* Overexpression of *OsHox32* leads to leaf rolling and semi-dwarf phenotype in rice, indicating that *OsHox32* plays pleiotropic effects on plant type architecture and leaf development in rice. *OsHox32* exhibited constitutive expression in different organs, with higher mRNA levels in the stem, leaf sheath, shoot apical meristems and young roots, suggesting a role in plant-type and leaf development. In addition, *OsHox32* mRNA levels were higher in light and lower in the dark, indicating that *OsHox32* may be associated with light regulation. Photosynthesis-associated and chlorophyll biosynthesis-associated genes were down-regulated to result in the reduction of photosynthetic capacity in *OsHox32-OV* lines. The expression of *YABBY* genes is up-regulated or down-regulated by *OsHox32*, suggesting that *OsHox32* may regulate the architecture of plant type and leaf development by controlling the expression of *YABBY* genes in rice.

## Methods

### Plant Materials

The rice variety Nipponbare (*Oryza sativa* L.ssp*. japonica*) and transgenic plants overexpressing *OsHox32* were used in this study. The transgenic plants were generated from Nipponbare using the *Agrobacterium-*mediated transformation method described by Hiei et al. (1994). Plants were grown in the experimental field at the Tianjin Agricultural Academy of Science, Tianjin, and in Lingshui, Hainan Province, China.

### Construction of the Overexpression Vector and Rice Transformation

*OsHox32* cDNA was amplified from 15-day-old seedlings by RT-PCR using specific primers: OHox32F: 5’ - TTAA*ggtacc*GAGAAGAAGGAGAAGGGTCG-3’ and OHox32R: 5’ - GAGC*tctaga*CTCAGACGAATGACCAGTTG-3’. The italic bases are recognition sites for restriction endonuclease enzymes. The resulting fragment was inserted into an empty pCAMBIA2300 binary vector with double CaMV 35S promoters to obtain the resultant vector. After confirmation by sequencing, the resultant vector was introduced into wild-type plants using the *Agrobacterium*-mediated transformation procedure described by Hiei et al. (1994).

### RT-PCR and Real-Time PCR Analysis

For analysis of gene expression in transgenic lines, RNA was isolated from 50-day-old leaves using Trizol reagent (Invitrogen, USA) and treated with DNase I (NEB, USA). For expression analysis of *OsHox32* in different organs, RNA was isolated from shoot apical meristems (SAM), young roots, mature leaf blades, leaf sheathes, stems of the first internode, and young panicles at different periods. For expression analysis of different development stages, penultimate leaves were collected at the desired stages for analysis of the expression of *OsHox32*. cDNA was synthesized from 2 μg total RNA using M-MLV reverse transcriptase (TaKaRa, Dalian, China). One microliter of cDNA was used for RT-PCR analysis with gene-specific primers. *OsHox32* amplification conditions were as follows: 2 min at 95 °C; followed by 32 cycles of 30 s at 94 °C, 30 s at 57 °C, 30 s at 72 °C; and then a final extension for 5 min at 72 °C. For *OsActin1*: 2 min at 95 °C; followed by 24 cycles of 30 s at 94 °C, 30 s at 60 °C, 30 s at 72 °C; and then a final step of 5 min at 72 °C. Three replicates were carried out for each reaction. Real-time PCR was performed using 1 μl cDNA and SYBR Green PCR master mix (Tiangen, Beijing, China) in a MyiQ2 two-color real-time PCR detection system (Bio-Rad, USA). The 2^-△△Ct^ method described by Livak (Livak and Schmittgen 2001) was used for the analysis of relative gene expression. Three replicates were performed for each reaction, and *OsActin1* was used as an internal control for the relative quantification of target gene expression. All primer sequences are provided in Additional file 1: Table S1. The amplification conditions were as follows: 2 min at 95 °C; followed by 40 cycles of 20 s at 95 °C, 30 s at 60 °C, and 30 s at 68 °C.

### Expression Analysis of *OsHox32* During Different Photoperiods

Strong wild type seedlings approximately 3 weeks old were transferred from natural fields to artificial climate cabinets (MMM, Climacell, Germany). Two photoperiods were used in the artificial climate cabinets with the following parameters: Long-day condition (LD), 15 h light and 9 h dark at 28 °C; short-day condition (SD), 9 h light and 15 h dark at 28 °C. After 22 days in artificial climate cabinets, RNA was isolated from the leaves once every 3 h for 24 h using Trizol solution (Invitrogen, USA). qRT-PCR was used to analyze the expression of *OsHox32* using the same method described as above.

### Histological Analysis and Leaf Rolling Index

Approximately 60-day-old leaves were collected from wild-type and transgenic plants and were fixed in an FAA solution (70 % ethanol, 5 % formaldehyde and 5 % acetic acid) for 48 h. After dehydration via ethanol series, the samples were infiltrated and embedded in paraffin. 10 μm thick sections were cut using a rotary microtome (Leica RM2235; Leica microsystems, Nussioch, Germany), stained with toluidine blue, and observed under a light microscope (eclipse 80i; Nikon, Tokyo, Japan). The width of leaves in the late booting stage was measured to calculate the leaf rolling index (LRI). The widest position at the middle of every leaf was measured. We measured the leaf width under natural rolling conditions (Ln) and while unfolding from rolling (Lw), and the LRI was calculated as LRI(%) = (Lw-Ln)/Lw × 100 %. The leaf area index was measured using a leaf area meter (CI-203 Area Meter, CID, Inc. USA).

### Measurement of Photosynthesis Indexes and Chlorophyll Content

In the field on a sunny morning, the net photosynthetic rate (Pn), stomatal conductance (Gs), intercellular CO_2_ concentration (Ci), transpiration rate (Tr), and water use efficiency (WUE) were measured in the penultimate and antepenultimate leaves of rice plants during the booting stage using a portable photosynthesis system equipped with an LED (light-emitting diode) light source 6400–02 (LiCor-6400; LiCor Inc. Lincoln, Nebraska, USA). Chlorophyll was extracted from 0.2 g fresh leaves at the booting stage using 80 % acetone. Briefly, leaves were cut into 3 mm pieces and immersed 80 % acetone for 48 h in the dark at 26 °C. The absorbance of the extract was measured using a spectrophotometer at A_645_ and A_663_. The chlorophyll a, chlorophyll b and total chlorophyll contents were determined by the method reported by Arnon (1949). A total of 10 individuals of the T_2_ transgenic line (*OsHox32-OV2*) and wild type plants, respectively, were assayed.

### The Subcellular Localization of *OsHox32*

The full region encoding of *OsHox32* without a stop codon was amplified from 30-day-old seedling cDNA by RT-PCR using gene-specific primers: G-Hox32F: 5’-ATA*gagctc*GAGAAGAAGGAGAAGGGTCG-3’ and G-Hox32R: 5’- CTG*tctaga*GACGAATGACCAGTTGACGA-3’. The italic bases are restriction endonuclease recognition sites. The amplified cDNA fragment was inserted into a pCAMBIA35S-GFP empty vector and fused in-frame with green fluorescent protein (GFP) to produce the resultant vector p35S::OsHox32*-*GFP. After confirmation by sequencing, the fused vector and empty vector were transiently transformed. To analyze tobacco epidermal cells, the fusion vector and empty vector were introduced into *the Agrobacterium tumefaciens* strain and injected into tobacco (*Nicotiana tabacum*) leaves by infiltration of the *Agrobacterium tumefaciens* strain. After culturing for 2–3 days, the tobacco epidermis was visualized using a laser confocal microscope (Nikon EZ-C1 Si laser confocal microscope, Japan). For onion transient expression analysis, the fusion vector p35S::OsHox32*-*GFP and empty vector were transformed into onion (*Allium cepa*) epidermal cells by gene bombardment (the Bio-Rad PDS-1000/He device, USA). Bombarded epidermal cells were incubated for 2–3 days at 25 °C in the dark. The cell layers were then examined using a laser confocal microscope (Nikon EZ-C1 Si laser confocal microscope, Japan).

### Phytohormone Treatments

Nipponbare (*Oryza sativa* L. ssp. *Japonica*) was planted in a greenhouse (28 °C, 14 h light, 10 h dark), and 4-week-old seedlings were subjected to different treatments. Phytohormone treatments were performed by spraying with IAA (0.1 mM), JA (0.1 mM), ACC (0.1 mM) and ABA (0.1 mM) solutions, and water was sprayed as a control. Leaves were collected for total RNA isolation to analyze *OsHox32* expression at 0, 1, 3, 6, 12 and 24 h, respectively.

## Additional file

Additional file 1: Table S1. Primers used in this study (XLS 19 kb)
